# Role of zeolite and palm fiber in modifying the strength of cement-treated soil

**DOI:** 10.1371/journal.pone.0313346

**Published:** 2024-11-05

**Authors:** Jili Qu, Lingqing Hu, Shouqian Wang, Wang Li, Jingyuan Yin, Xu Yang, Siyu Yao, Andrian Batugin, Cristea Lavinia Mihaela

**Affiliations:** 1 Department of Geotechnical Engineering, Faculty of Civil Engineering, Kashi University, Kashi, Xinjiang, China; 2 Department of Civil Engineering, Faculty of Environment and Architecture, University of Shanghai for Science and Technology, Shanghai, Shanghai, China; 3 Faculty of Architectural Engineering, Kashi University, Kashi, Xinjiang, China; 4 Faculty of Life and Geography, Kashi University, Kashi, Xinjiang, China; 5 Department of Mining Safety and Ecology, School of Mining Safety, National University of Science and Technology (MISIS), Moscow, Moscow, Russia; 6 Department of Accounting, Bucharest University of Economic Studies, Bucharest, Bucharest, Roma; 7 Department of Civil Engineering, School of Environment and Architecture, University of Shanghai for Science and Technology, Shanghai, Shanghai, China; Texas A&M University System, QATAR

## Abstract

This paper aimed to research the role of zeolite and palm fiber in changing strength of cement-treated soil. The unconfined compressive strength (UCS) testwas mainly used to study the changes of strength of cement-treated soil by varied content of cement, zeolite, palm fiber under different curing period. The optimum substitution rate of zeolite for cement was researched for the purpose of increasing of strength of cement-treated soil and low carbon and environment protection, while the optimum content of palm fiber was explored for improving of strength and ductility of cement-treated soil by means of analytic software. Finally, the multiple regression technology was used to try to capture the relationship between strength of cement-treated soil and content of zeolite and palm fiber. The results show that the cement, zeolite, palm fiber and curing age have strong effect on the UCS of cement-treated soil.

## Introduction

Shanghai is located in the east of China, the center of the Yangtze River Delta, with weak soil, high water content and strong compressibility. In general, corresponding reinforcement treatment should be carried out before construction. Traditional reinforcement methods such as lime, cement and gypsum do not conform to the concept of low-carbon and sustainable development due to environmental pollution and high cost [1–6]. In 2020, global CO_2_ emissions from cement production alone amounted to 1.23 billion tonnes [7, 8]. In order to achieve China’s goal of "2030 carbon peaking and 2060 carbon neutrality", it is imperative to develop new reinforcement methods that are in line with the concept of low-carbon and sustainable development. Zeolite is a material with volcanic ash activity [9], and the zeolite can be ground into powder and reacted with slaked lime and water to produce products with gelling properties, so the use of zeolite powder instead of part of cement to reinforce Shanghai clay is an innovation in this study. In addition, palm fiber was also selected as a reinforcement material for this study because of its environmental protection, low cost, easy availability and can greatly improve soil strength and reduce brittleness when incorporated into soil. Since the addition of an appropriate amount of cement to Shanghai clay greatly increases the strength of the soil, this project is based on cement-treated soil.

Cement-treated soil is an artificial mixed material formed by mixing cement with soil after adding water. Cement clinker in cement-treated soil is mainly composed of four minerals [10, 11], namely tricalcium silicate 3CaO·SiO_2_(C3S), dicalcium silicate 2CaO· SiO_2_ (C2S), tricalcium aluminate 3CaO· Al_2_O_3_(C3A), tetracalcium ferroaluminate 4CaO· Al_2_O_3_· Fe_2_O_3_(C4AF). Tricalcium silicate C3S accounts for the highest proportion, followed by Dicalcium Silicate C2S, and the content of Tricalcium Aluminate C3A and Tetracalcium Ferroaluminate C4AF accounted for relatively small. The results of previous research [12–14] showed that the variety of cement, cement content, curing conditions, curing age, soil properties and moisture content are all important factors affecting the strength of cement-treated soil. Horpibulsuk et al. [15–17] introduced the water-cement ratio as a variable when the amount of cement was fixed, and studied the unconfined compressive strength, triaxial compression characteristics, consolidation characteristics, and microstructure of cement-treated soil. It was found that the water-cement ratio plays a controlling role in the strength and deformation characteristics of cement-treated soil, so the water-cement ratio was proposed to predict the strength of cement-treated soil and gave a formula for predicting the strength of cement-treated soil. Wang et al. [18] introduced the comprehensive parameters of water-cement ratio and initial density composition of soil samples to predict the strength of cement-treated soil, and the prediction results were more accurate than the actual test results with single water-cement ratio as a parameter. Zhu et al. [19, 20] through a series of mechanical tests of cement-treated soil, obtained the relationship between cement content, curing age and compressive strength of cement-treated soil, and concluded that the optimal cement content was 7%~20%, the early strength of cement-treated soil increased rapidly, and its strength was 60%~70% of 28 days when the curing period was 7 days, and 80%~90% of the strength of 90 days in 28 days, which could be regarded as the final strength after the hydration and hardening of cement-treated soil. In addition to reinforcing the soil with plain cement, researchers added fly ash, metakaolin, slag rubber, nanomaterials, graphene oxide and other materials [21–32] to the cement-treated soil to form composite cement-treated soil, and studied the properties of composite cement-treated soil, and the mechanical properties of composite cement-treated soil were improved to a certain extent compared with plain cement-treated soil. Researchers [33–43] added synthetic fiber and natural plant fiber to cement-treated soil, and found that fiber cement-treated soil was greatly improved in terms of ductility, compressive strength and durability compared with ordinary cement-treated soil.

Natural zeolite [44, 45] is mainly composed of SiO_2_, Al_2_O_3_, H_2_O and other components, and the general chemical formula is: [M2(I.)M(II)] O· Al_2_O_3_·nSiO_2_·mH_2_O. M(I) and M(II) are metals with valence states of monovalent and bivalent (often sodium, potassium, calcium and other metals), and n is the ratio of Si to Al in zeolite, and m is the number of water molecules. The basic structural units of zeolite crystals are silicon-oxygen tetrahedron and aluminum-oxygen tetrahedron, which form the skeleton of zeolite. The skeleton of zeolite is a frame-like structural skeleton, with many interconnected cavities and pores inside, allowing molecules of a certain size to move freely in it, which determines that zeolite has certain adsorption characteristics. Wang et al. [46, 47] used the adsorption properties of zeolite to add zeolite to radioactive cement-treated soil materials, and found that the addition of zeolite can stabilize the metal ions in cement-treated soil, effectively reduce the radioactivity of cement-treated soil, and the smaller the particles of zeolite, the stronger its shielding radioactivity. Yang [48] incorporated zeolite into concrete and found that zeolite powder had the best moisture absorption performance after heat treatment at 500°C for 2h, and the best moisture absorption rate reached 13.6%. SIO_2_ and Al_2_O_3_ in zeolite account for 80% of the composition, and its volcanic ash activity is between silica ash and fly ash [49], which is better than fly ash. Li et al. [50] added zeolite powder to high-strength concrete and found that the volcanic ash reaction of SiO_2_ and Al_2_O_3_ in zeolite powder made the structure more solid, which could effectively inhibit the alkaline aggregate reaction of concrete and improve the durability of concrete. Gui et al. [51] studied the effect of zeolite powder on the hydration reaction mechanism of sulfaluminate cement. The test data show that the fluidity of zeolite sulfaluminate cement decreases, and the setting time shows a trend of first increasing and then decreasing with the increase of zeolite content, and the early strength of cement slurry is significantly increased under the content of 5~15% zeolite, which is conducive to the formation of ettringite. Jafarpour et al. [52] used the consolidated undrained triaxial test to study the mechanical properties of zeolite cement reinforced sand, and the friction angle and cohesion in zeolite cement sand increased with the increase of zeolite content under an appropriate amount of zeolite substitution rate. China is a country rich in zeolite resources, with proven reserves of 10 billion tons, and the predicted natural zeolite prospect can reach 50 billion tons, ranking first in the world [50], and zeolite is cheap and easy to mine, and studying the relevant characteristics of zeolite on cement-based materials is of great significance to the carbon reduction of the cement industry.

Palm fiber is a natural plant polymer material [53], which has the advantages of large yield, renewable output, convenient access, strong natural anti-corrosion function, and strong moisture absorption ability. Palm fiber has a large number of fine hollow reinforced single fibers inside, which has a good unidirectional tensile ability. In water conservancy engineering, geotechnical engineering and other fields, it can be mixed with soil, concrete and other materials to form composite materials as a strength enhancer, which plays a role in replacing mineral resources. Qu et al. [54] studied the strength characteristics of palm reinforcement of Shanghai clay, and obtained the better palm reinforcement rate and palm fiber reinforcement size of palm fiber to Shanghai clay. The results showed that the cohesion of the soil sample increased greatly after the addition of palm fiber, but the internal friction angle did not change significantly. The palm fiber size of 6 mm x 12mm had better shear resistance than 6mm x 6mm and 6mm x 18mm, and the optimal fiber content is 0.6%, and the soil had better ductility and deformation resistance. Huang et al. [55] explored the influence of palm fiber on soil strength and deformation from the perspective of compressive strength and energy absorption. The test results showed that the 0.5% palm fiber content and 20mm fiber length are a more suitable ratio, and under the optimal moisture content, the palm fiber has the best reinforcement effect on the soil. Ozerkan et al. [56] studied the mechanical properties and durability of palm fiber reinforced mortar and found that the 1%-2% palm fiber content was a reasonable and effective amount, and the durability and drying shrinkage resistance of the mixture were improved. Lertwattanaruk et al. [57] applied palm fiber in the field of construction and housing, and studied the effect of adding 5%~15% palm fiber to cement-based materials on the properties of cement-based materials. The results showed that palm fiber cement board had a low thermal conductivity, which could reduce the energy exchange between indoor and outdoor and improve the energy efficiency of the indoor building environment. Cao et al. [58] studied the effect of palm fiber and xanthan gum on the compressive strength of Shanghai clay. The results showed that the addition of palm fiber and xanthan gum could improve the compressive strength of the soil, but the xanthan gum would increase the brittleness of the soil, and the palm fiber could improve the ductility of the soil, and the optimal incorporation ratio of xanthan gum and palm fiber to improve Shanghai clay is given.

In this paper, the strength characteristics of cement-treated soil with different zeolite and palm fiber contents would be studied, so as to expand the scope of reinforcement materials and engineering options, and provided theoretical and practical incremental knowledge for Shanghai cohesive soil reinforcement.

## Material and method

### Material

#### Soil

**Fig 1:**
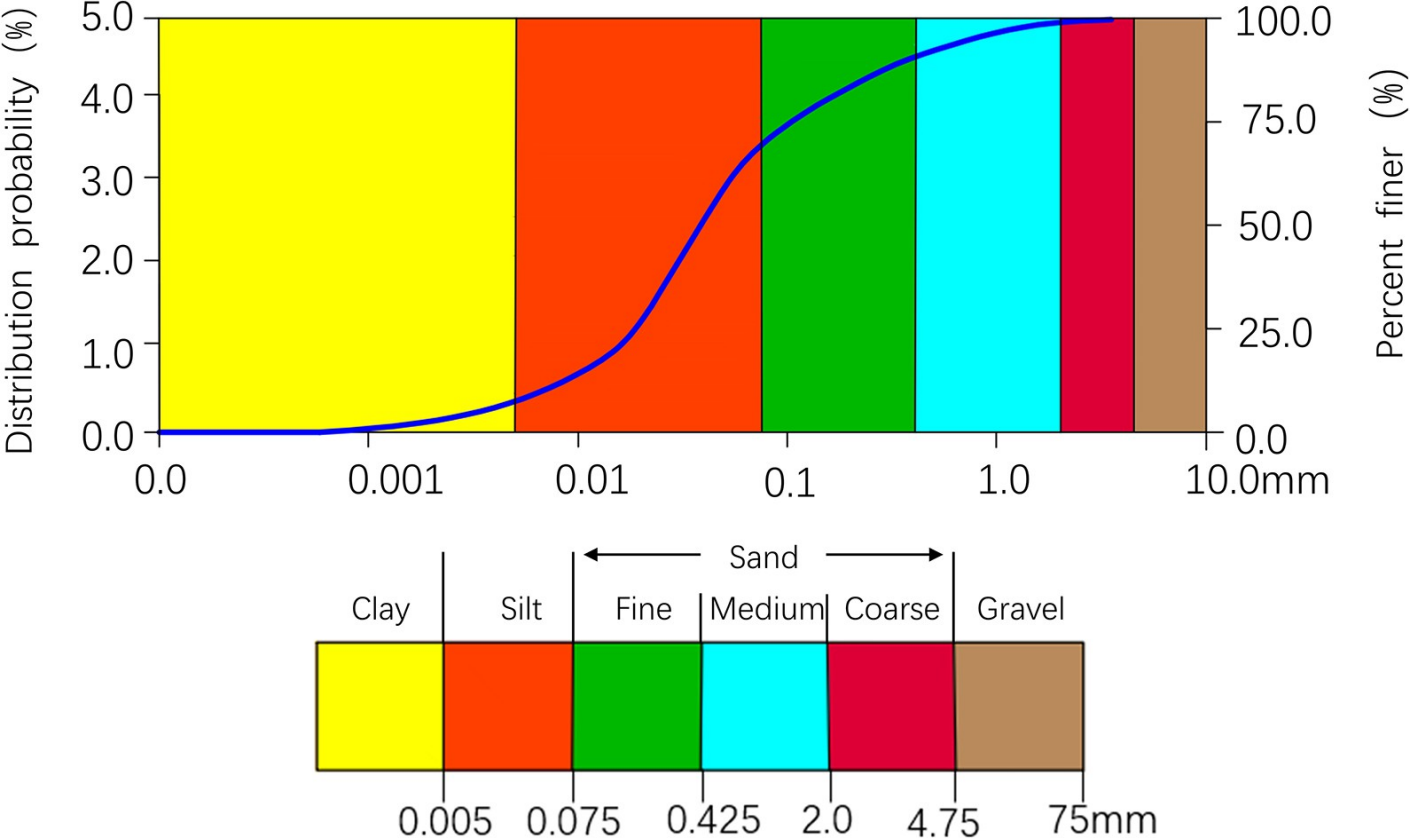
Particle size distribution of test soil (provided by Malvern Instruments Ltd –www.malvern.com).

The test soil samples were taken from an open land next to the Yangpu Riverside in Shanghai, and the soil was selected 1.5 m below the surface to avoid the influence of artificial soil fill on the surface. The soil extraction site is situated 150 meters away from the bank of the Huangpu River tributary. Following retrieval of the soil sample, it underwent air drying and pulverization using a pulverizer. The soil was then sifted through a 2 mm sieve and securely sealed for future utilization. Upon acquiring the test soil, a series of tests including the liquid and plastic limit test (ASTM D4318-10) and the compaction test (ASTM D698) were conducted. Consequently, the physical and hydrological characteristics of the soil sample were documented in Table 1, while its chemical properties were outlined in Table 2. Furthermore, the particle size distribution of the soil sample was clearly illustrated in Fig 1.

**Table 1:** Basic physical and hydrological properties of soil used.

Optimum water content (%)	Maximum dry density/ (g/cm^3^)	Plastic limit/ (%)	Liquid limit/ (%)	Plasticity index
20	1.69	23	45	22

**Table 2:** The chemical composition of the test soil*.

SiO_2_	Al_2_O_3_	Fe_2_O_3_	CaO	K_2_O	MgO	Na_2_O	TiO_2_	Others
65.93	13.40	7.03	4.57	3.20	2.49	1.38	1.15	0.85

*Note: provided by Shanghai Zhongzheng Analysis and Test Technology Service Center.

#### Zeolite

**Fig 2:**
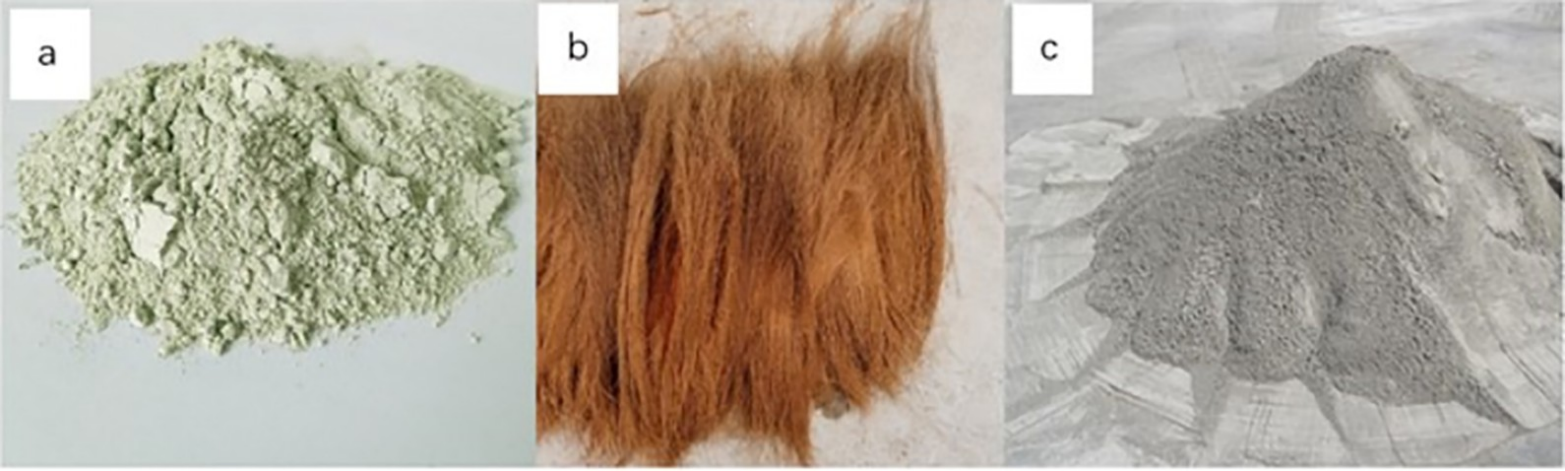
Photgraphs for zeolite powder (a), palm fiber (b) and cement (c).

The zeolite used in the test is all natural clinoptilolite zeolite powder produced by Chifeng Hengyuan Mineral Products Co., Ltd., see Fig 2A. The particle size of zeolite powder is 600 mesh (sieve size: 0.023mm), and the chemical composition of zeolite powder was shown in Table 3.

**Table 3:** Chemical composition of zeolite powder.

composition	SiO_2_	Al_2_O_3_	Fe_2_O_3_	CaO	MgO	SO_3_	K_2_O	Na_2_O
Content (%)	68.59	11.47	1.07	4.33	0.84	0.06	1.15	0.80

#### Palm fiber

The palm fibers used in the experiment were all taken from palm trees planted on Chongming Island in Shanghai, as shown in Fig 2B. After taking it back, the palm was washed, placed in a cool and ventilated place, and naturally air-dried, and then the palm was cut to the filamentous length required for the test, and the physical characteristics of the palm fiber is shown in Table 4.

**Table 4:** Physical properties of palm fiber.

Diameter (mm)	density (g/cm^3^)	Breaking strength (MPa)	Elastic module (GPa)	Elongation at break (%)
0.2~0.9	1.28	125~218	0.54~1.06	15~23

#### Cement

The cement used in the test was all conch brand cement, see Fig 2C. The cement label is P.O 42.5, and the properties of cement met the relevant national standards, and the chemical composition of cement is shown in Table 5.

**Table 5:** Chemical composition of cement.

ingredient	SiO_2_	Al_2_O_3_	Fe_2_O_3_	CaO	MgO	SO_3_	K_2_O	Na_2_O
Content (%)	21.05	4.55	3.13	62.17	2.63	3.10	0.28	0.32

### Method

#### Specimen preparation and curing

No permits for all test sites access were required because the instruments, the equipment and the test grounds were our own.

The specimen preparation could be divided into 4 steps. First, the pure soil was theoretically configured according to the optimal water content of 20%, and the actual is 21% to offset the loss of water during the preparation of the soil sample. We used an electric mixer to stir the pure soil well and sealed it with plastic wrap for 24h to spread the moisture in the sample evenly. Second, water and cement were mixed based on 0.5 ratio of water to cement, stirred evenly. Noted the weight of zeolite and palm fiber was also counted as the weight of cement in calculating the ratio of water to cement. Third, pure soil sample made in the first step and sample with only cement (may include zeolite and /or palm fiber) made in the second step were fully mixed and stirred evenly with electric mixer. Fourth, a cylindrical test block of 39.1mm ×80mm was made according to the dry density of 1.69g/cm3 with the soil-cement-water mixture made in the third step.

**Fig 3:**
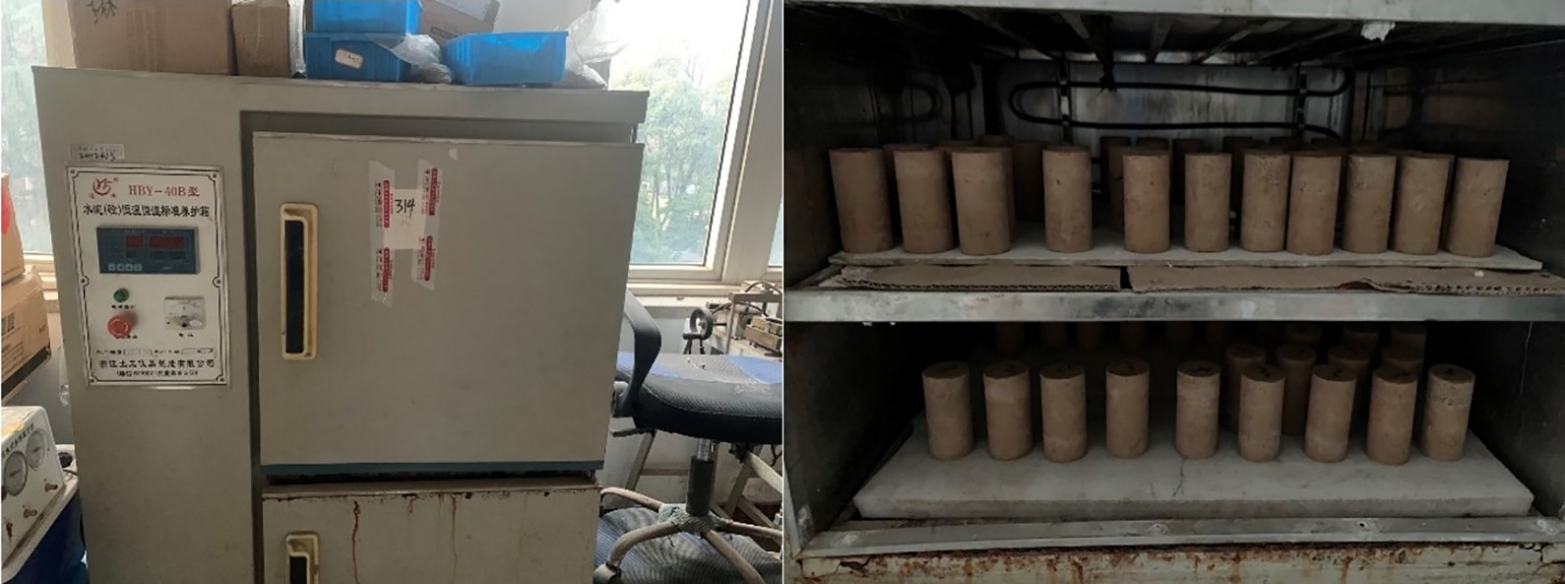
Standard curing chamber and partial curing test block.

According to the research results of predecessors, the soil sample mix ratio was designed with cement content (mass fraction relative to soil) of 10%, 15% and 20%, zeolite replacement rate (mass fraction relative to cement) of 10%, 30%, 50%, 70% and 90%, palm fiber content (mass fraction relative to soil) 1%, 1.5% and 2%, and the water-cement ratio of soil samples were 0.5. According to the designed mixing ratio, all samples were made one by one, numbered and wrapped with plastic film. Then we removed the plastic wrap after 24h and put it into a standard curing box. The curing temperature was 20±2°C and the humidity was 95%. The test blocks were cured to the required age. The standard curing tank and some curing test blocks are shown in Fig 3.

#### Test

The instrument used in the compaction test of plain soil was the I-1 light compaction instrument produced by Zhejiang Geotechnical Instrument Manufacturing Co., Ltd. This instrument was used to determine the maximum dry density and optimal water content of plain soils. The instrument used in the liquid and plastic limit test was the STYS-1 digital liquid and plastic limit joint tester produced by Zhejiang Geotechnical Instrument Manufacturing Co., Ltd. The instrument used in the cement unconfined compressive strength test was WDW-Y300D microcomputer-controlled automatic pressure testing machine produced by Jinan Zhongzheng Testing Machine Company, and the maximum test force of the testing machine was 300KN. The unconfined compressive strength test of cement-treated soil was carried out with reference to the Technical Rules for the Construction of Highway Pavement Base Layers (JTG/T F20-2015) [59], and the axial pressure speed of the specimen was 1mm/min. The failure criteria were: when the dynamometer reading peaked, continued, and stopped the test after 3~5% strain or the test was carried out until 20% strain. Multiple regression analysis was carried out by the software SPSS. Each experiment was repeated three times, with the average value as the experimental result.

#### Research protocol

1. Technology roadmap
2. Detailed test protocol

**Fig 4:**
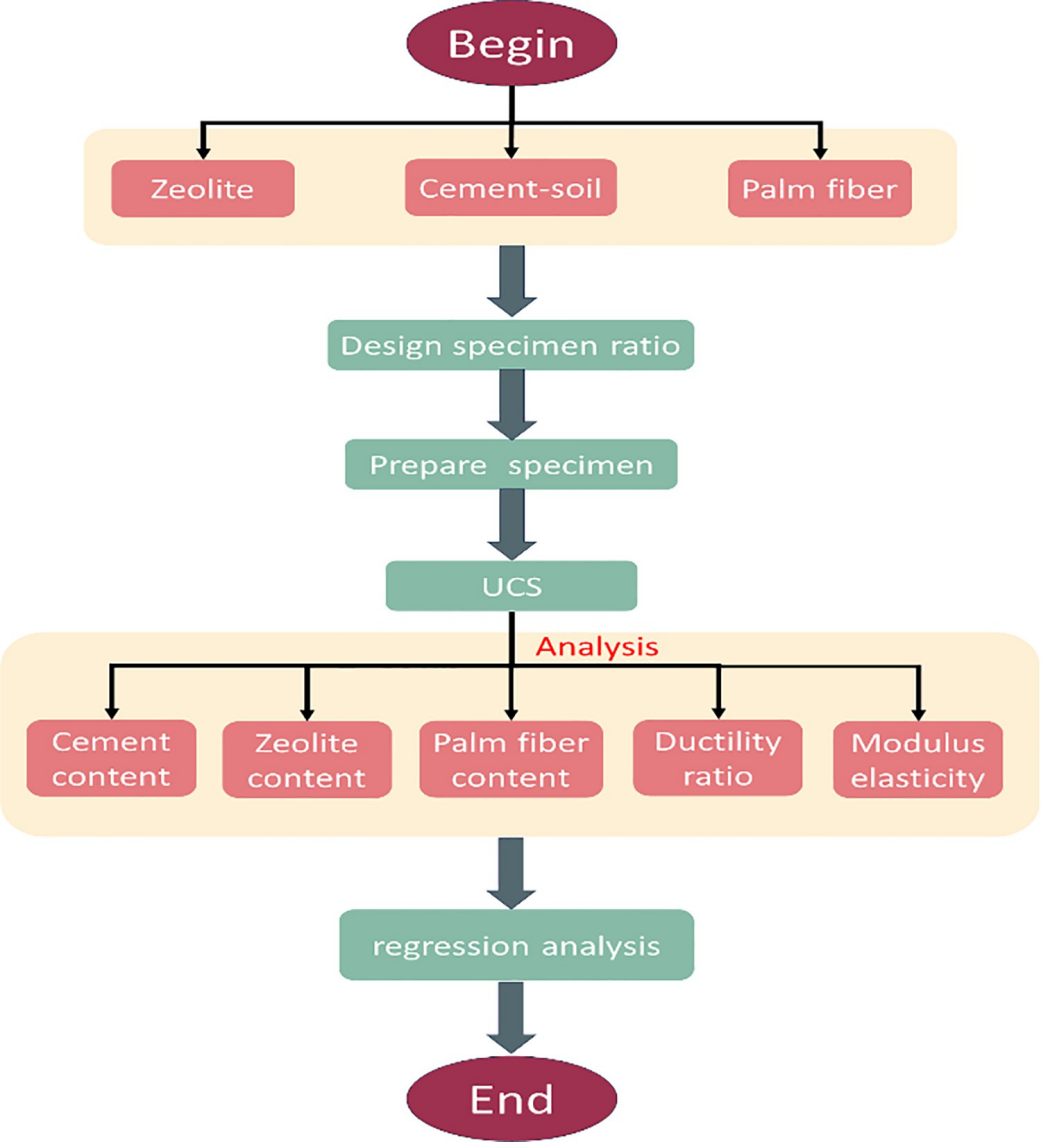
Roadmap of research.

The roadmap of the research is shown in Fig 4:

The detailed test protocol is shown in Table 6. C represents cement, Z represents zeolite, F represents palm fiber, and the following numbers represent the content of this admixture. For example, C10-Z30-F0 represents that cement accounts for 10% of the soil sample mass, zeolite accounts for 30% of the cement mass, and palm fiber accounts for 0% of the soil sample mass.

**Table 6:** The detailed test plan.

Group name	Specimen no	Cement (%)	Zeolite %)	Palm fiber %)
S+C	C10-Z0-F0	10	0	0
	C15-Z0-F0	15	0	0
	C20-Z0-F0	20	0	0
S+C10+Z	C10-Z10-F0	10	10	0
	C10-Z30-F0	10	30	0
	C10-Z50-F0	10	50	0
	C10-Z70-F0	10	70	0
	C10-Z90-F0	10	90	0
S+C15+Z	C15-Z10-F0	15	10	0
	C15-Z30-F0	15	30	0
	C15-Z50-F0	15	50	0
	C15-Z70-F0	15	70	0
	C15-Z90-F0	15	90	0
S+C20+Z	C20-Z10-F0	20	10	0
	C20-Z30-F0	20	30	0
	C20-Z50-F0	20	50	0
	C20-Z70-F0	20	70	0
	C20-Z90-F0	20	90	0
S+C10+F	C10-Z0-F1	10	0	1
	C10-Z0-F1.5	10	0	1.5
	C10-Z0-F2	10	0	2
S+C15+F	C15-Z0-F1	15	0	1
	C15-Z0-F1.5	15	0	1.5
	C15-Z0-F2	15	0	2
S+C20+F	C20-Z0-F1	20	0	1
	C20-Z0-F1.5	20	0	1.5
	C20-Z0-F2	20	0	2
S+C10+Z10+F	C10-Z10-F1	10	10	1
	C10-Z10-F1.5	10	10	1.5
	C10-Z10-F2	10	10	2
S+C10+Z30+F	C10-Z30-F1	10	30	1
	C10-Z30-F1.5	10	30	1.5
	C10-Z30-F2	10	30	2
S+C15+Z10+F	C15-Z10-F1	15	10	1
	C15-Z10-F1.5	15	10	1.5
	C15-Z10-F2	15	10	2
S+C15+Z30+F	C15-Z30-F1	15	30	1
	C15-Z30-F1.5	15	30	1.5
	C15-Z30-F2	15	30	2
S+C20+Z10+F	C20-Z10-F1	20	10	1
	C20-Z10-F1.5	20	10	1.5
	C20-Z10-F2	20	10	2
S+C20+Z30+F	C20-Z30-F1	20	30	1
	C20-Z30-F1.5	20	30	1.5
	C20-Z30-F2	20	30	2

## Results and discussion

### Effect of different factors

**Fig 5:**
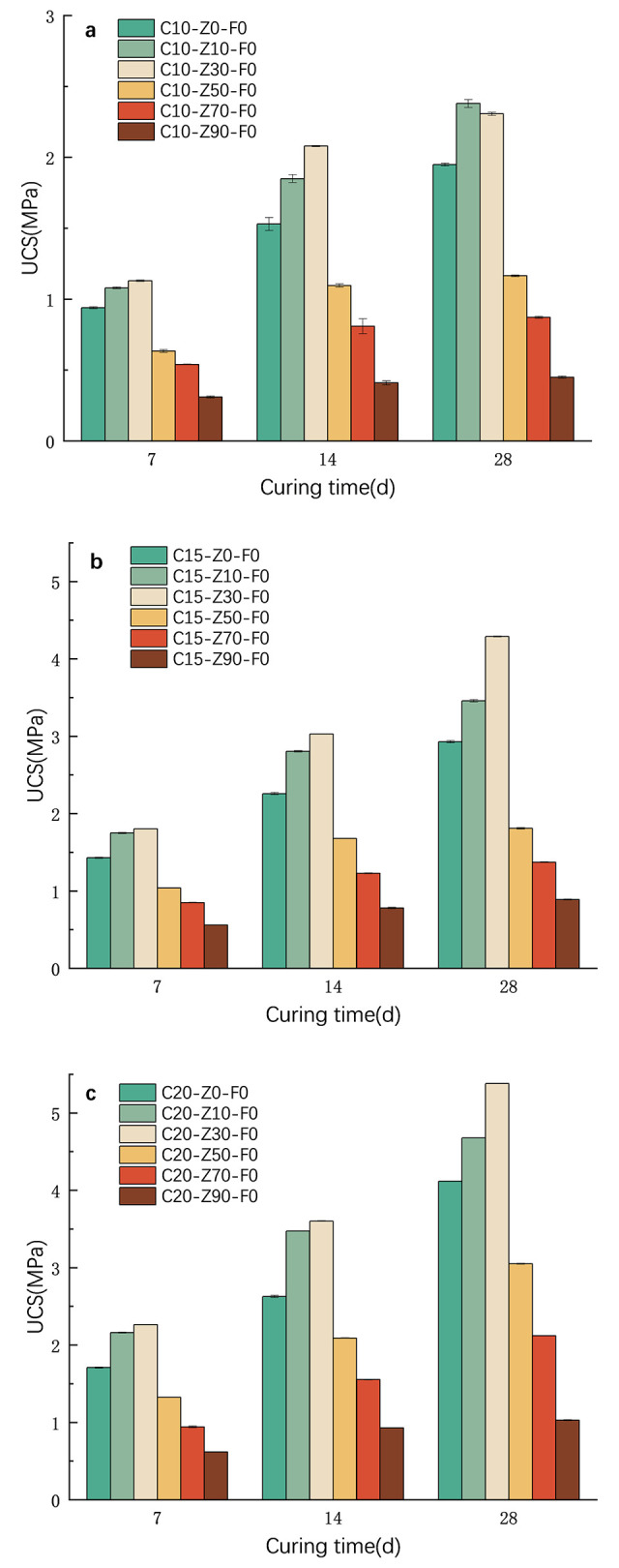
Relationship between strength of cement-treated soil and content of zeolite and curing time under different cement content. (a: 10%; b:15%; c:20%). Error bars on the top of bar chart.

Fig 5 shows the relationship between strength of cement-treated soil and content of zeolite and curing time under different cement content (a: 10%; b:15%; c:20%). Note that the content of cement is the percentage of weight of cement relative to soil mass, while the content of zeolite is the percentage of weight of zeolite relative to cement mass. The curing period is 7 days (7d), 14 days (14d) and 28 days (28d).

#### Effect of curing period

Fig 5A–5C are a column chart of the unconfined compressive strength of 0, 10, 30, 50, 70 and 90% zeolite replacement rates at a curing age of 7 days, 14 days and 28 days, respectively. The cement content of a, b, c is 10, 15 and 20%, respectively. It can be seen from Fig 5 that the unconfined compressive strength of plain cement-treated soil (only soil + cement) and zeolite cement-treated soil increases with the increase of curing age. The growth rate of zeolite cement strength from the 7 days to 14 days of curing age is much faster than that of 14 days to 28 days, which indicates that the strength of zeolite cement-treated soil is formed relatively quickly in the early stage, and the strength growth rate slows down after 14 days. When the zeolite replacement rate is 10% and 30%, the strength of zeolite cement-treated soil is higher than that of plain cement-treated soil regardless of age. However, when the zeolite replacement rate is greater than or equal to 50%, the strength of zeolite cement-treated soil is lower than that of plain cement-treated soil regardless of age. At 10% cement content (a), the strength of C10-Z10-F0 specimens increased from 1.10MPa to 2.30MPa from 7d to 28d, an increase of 109%. At 15% cement content (b), the strength of C15-Z30-F0 specimen increased from 1.80MPa to 4.40MPa from 7d to 28d, an increase of 144%. At 20% cement content (c), the strength of C20-Z30-F0 specimens increased from 2.60MPa to 5.40MPa from 7d to 28d, an increase of 107%. In addition to C10-Z30-F0 (also close to the highest point at 28d), it can be seen that regardless of the cement content, when the zeolite replacement rate reaches 30%, the strength is the highest and the effect is best.

#### Effect of zeolite

Regarding the strength of cement-treated soil, the most concerned and generally referenced in engineering design is the 28-day strength. Therefore, the following focuses on the analysis of the 28-day strength of zeolite cement-treated soil. As shown in Fig 5(A), when the curing period is 28 days, the unconfined compressive strength of 10%, 30%, 50%, 70% and 90% zeolite cement-treated soil with 10% cement content is 1.950, 2.380, 2.307, 1.165, 0.872 and 0.450MPa, respectively. The zeolite replacement rate is 10% and 30%, which strengthens the strength of cement-treated soil. The peak value of the unconfined compressive strength of zeolite cement-treated soil occurred when the zeolite replacement rate was 10%, and its strength was 1.22 times that of the strength of plain cement-treated soil, which increased by 22%. When the substitution rate is 30%, its strength is 1.18 times that of plain cement-treated soil, which is increased by 18%. This number is very close. In Fig 5B, 5C, regardless of the curing age of 7d, 14d or 28d, and regardless of whether the cement content is 15% or 20%, the unconfined compressive strength of the specimen is the largest when the zeolite replacement rate is 30%. In view of this, for ease of use and memory, it may be considered that a 30% zeolite replacement rate has the highest strength, regardless of the cement content and curing age.

In addition, Fig 5 also shows that when the zeolite replacement rate exceeds 50%, the more zeolite, the lower the strength. Because no tests were set between 30% and 50%, the authors do not know exactly where the cut-off point for strength turns from increasing to decreasing. However, at least it can be considered that the strength increases before 30% zeolite replacement rate. The strength decreases with the increase of zeolite after 50%. And this has little to do with curing age and cement content.

#### Effect of cement

Compare Fig 5A–5C (cement content 10%, 15%, 20%, respectively), regardless of the curing age and the zeolite replacement rate, the strength of the specimen increases as the cement content increases. In our experiment, the cement content was 20%, the zeolite replacement rate was 30%, and the maximum strength of the specimen reached 5.4MPa at the curing age of 28 days, as shown in Fig 5C. It can be seen that the content of cement directly determines the strength of cement-treated soil. But too much cement is not good for low-carbon environmental protection and sustainable development, which is why zeolite is used instead of cement without reducing strength. At the curing age of 28 days, the strength of C20-Z30-F0 and C10-Z30-F0 samples was 5.4 and 2.3MPa, respectively, and the former increased by 135% over the latter.

### Unconfined compressive strength change rate

In order to further understand the effect of zeolite substitution rate on the strength of plain cement-treated soil, use the Formula (1) to calculates the change rate of unconfined compressive strength of zeolite cement-treated soil.

$$R=\frac{q_{u(s-c-z)}-q_{u(s-c)}}{q_{u(s-c)}}$$ (1)

**Fig 6:**
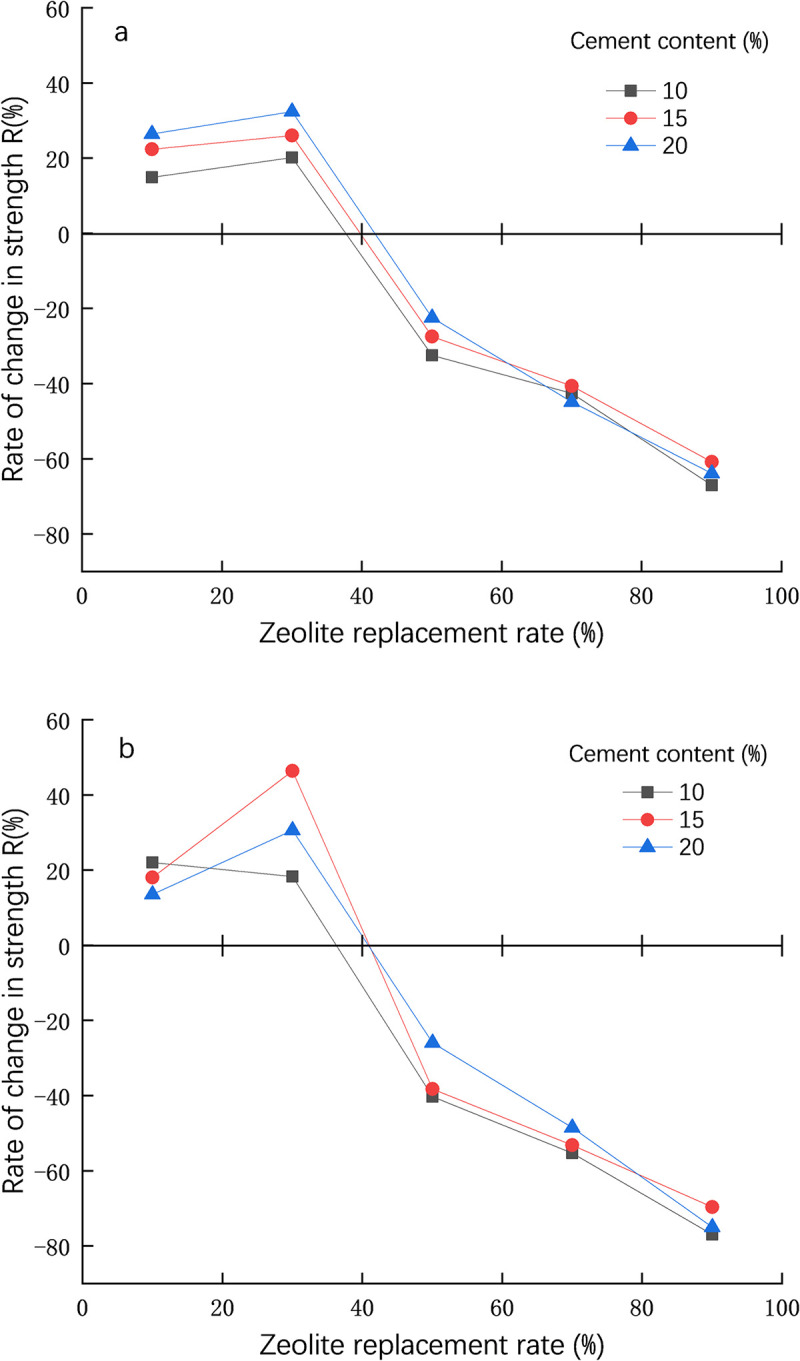
Variation rate of strength of zeolite cement-treated soil. a: 7d, b: 28d.

Where *R* is the change rate of unconfined compressive strength of cement-soil;

q_u(s-c-z)_ is the unconfined compressive strength of cement-treated soil after adding zeolite;

q_u(s-c)_ is the unconfined compressive strength of cement-treated soil before adding zeolite;

Fig 6A shows the unconfined compressive strength change rate (R) of the specimen with different cement content and different zeolite replacement rate at 7d. When the zeolite replacement rate is from 30% to 50%, if the strength change rate is assumed to be linear, the corresponding zeolite content can be directly found when the strength change rate is 0% (The intersection of the curve and the horizontal axis). The authors call this equal strength zeolite replacement rate (Zr). Fig 6A shows that when the curing age is 7d, the zeolite replacement rate is 30% and the cement content is 20%, the strength change rate R is the largest (35%), and then gradually decreases with the increase of zeolite replacement rate. At this time, the equal strength replacement rate (Zr) is about 43%. When the zeolite replacement rate is less than this value, the strength change rate is positive, and vice versa. Zr = 43% is the critical value of zeolite replacement rate at 7d curing age. Under the same conditions, the critical values of zeolite replacement rate at cement content of 10 and 15% are 37% and 40%, respectively.

Fig 6B shows the unconfined compressive strength change rate (R) of the sample with different cement content and different zeolite replacement rates at 28d. Fig 6B shows that when the curing age is 28 days, the zeolite replacement rate is 30% and the cement content is 15%, the strength change rate R is the largest (47%), and then gradually decreases with the increase of zeolite replacement rate. At this time, the iso-strength replacement rate (Zr) is about 40%. When the zeolite replacement rate is less than this value, the strength change rate is positive, and vice versa. Zr = 40% is the critical value of zeolite replacement rate at 28 days of curing age. Under the same conditions, the critical values of zeolite replacement rate at cement content of 10 and 20% are 37% and 40%, respectively. It is generally recommended that in actual use, regardless of the cement content and the curing period, the zeolite replacement rate cannot exceed the critical value (Zr = 40%).

### Stress-strain relationship of zeolite cement-treated soil

**Fig 7:**
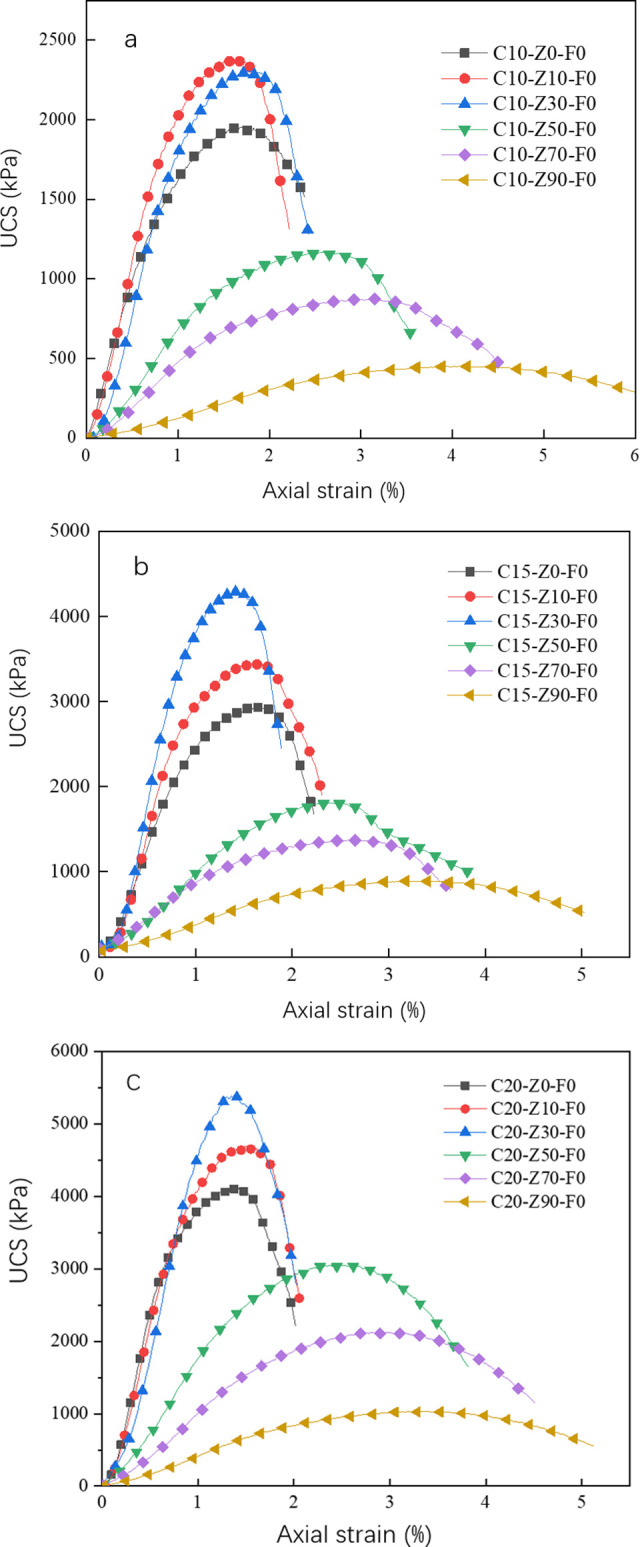
Relationship between stress and strain of the sample (28d). Cement: a 10%, b 15%, c20%.

Fig 7A–7C are the stress-strain relationship of zeolite cement-treated soil with 10%, 15% and 20% cement content at a curing age of 28 days, respectively. The overall observation of a, b and c shows that no matter what kind of ratio of zeolite substitution rate and cement content, the unconfined compressive strength of zeolite cement-treated soil is enhanced with the increase of axial strain, and when the peak compressive strength is reached, the compressive strength of zeolite cement-treated soil decreases with the increase of axial strain. The stress-strain curves are all in the shape of a "concave downward parabola". In Fig 7A, the corresponding strains (failure strains) of C10-Z10-F0 and C10-Z30-F0 specimens at maximum strength are 1.5% and 1.6%, respectively, which is slightly lower than the failure strain of C10-Z0-F0 (about 1.65%) of plain cement-treated soil. Failure strains of C10-Z50-F0, C10-Z70-F0 and C10-Z90-F0 were 2.5, 3.1 and 4.5%, respectively, which far exceeded that of plain cement-treated soil. It seems to be a law that the higher the peak strength, the smaller the failure strain, and the smaller the peak strength, the greater the failure strain. As the zeolite content is less than 30%, in the strength rising section, the strength growth rate is higher than that of plain cement-treated soil. Fig 7B, 7C show a similar pattern. Compared with the maximum strength of different cement contents, namely C10-Z30-F0 (a), C15-Z30-F0 (b), and C20-Z30-F0 (c), their failure strains were 1.6%, 1.45% and 1.25%, respectively. This data once again shows the conclusion that regardless of the zeolite content and cement content, the greater the peak strength, the smaller the failure strain. As zeolite is added to cement-treated soil, and its unconfined compressive strength increases first and then decreases with the increase of zeolite replacement rate, and an appropriate amount of zeolite content can improve the strength of zeolite cement-treated soil, but too much of the zeolite content will result in insufficient strength due to lack of cement hydration products. Regardless of the cement content, the zeolite replacement rate required for its maximum strength is basically maintained at about 30%. At the same time, zeolite powder not only improves the strength of cement-treated soil, but also has an impact on the ductility and brittleness of cement-treated soil.

### Effect of palm fiber on zeolite cement-treated soil

In order to improve the cracking and brittleness failure of cement-treated soil, plant fiber-palm fiber was added to zeolite cement-treated soil to improve the mechanical behavior of zeolite cement-treated soil. Palm fiber is green, low cost, and fully in line with the concept of low-carbon environmental protection. In this paper, 10% and 30% zeolite substitution rates and 15% cement content were selected to explore the effects of palm fiber on the mechanical properties of zeolite cement-treated soil. According to the research results in Ozerkan [56] and Cao Zhimin [58], 1%, 1.5% and 2% palm fiber content were selected as variables, and the length of palm fiber was 12±2mm.

#### Influence of palm fiber content

**Fig 8:**
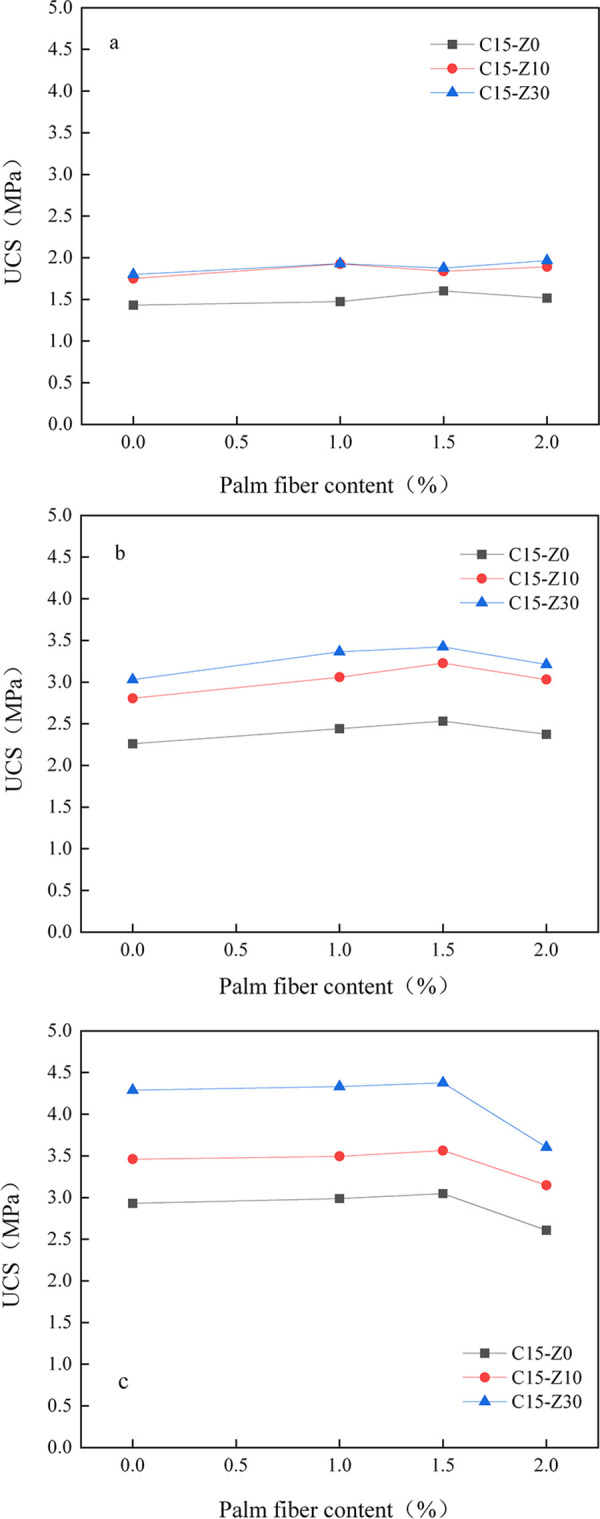
Relationship between unconfined compressive strength and palm fiber content of specimen. Curing period: a 7d; b 14d; c 28d.

Fig 8A shows the relationship between the unconfined compressive strength of the specimen and the palm fiber content when the curing period is 7 days. When 1%, 1.5% and 2% palm fiber are added to zeolite cement-treated soil, its strength is higher than 0% fiber. With the increase of palm fiber content, the peak compressive strength of C15-Z0 specimen first increased and then decreased, and the maximum peak strength appeared when the palm fiber content was 1.5%, and its compressive strength increased by 11.9% compared with that of 0% fiber sample. C15-Z10 and C15-Z30 showed a change pattern of first increasing and then decreasing and then increasing with the content of palm fiber, and the peak compressive strength occurred at 1% and 2% palm fiber content, respectively. At this time, there is no obvious regularity between palm fiber content and strength. The reason for this phenomenon may be that the cement, zeolite, palm fiber and soil particles in the cement-treated soil do not fully function during the 7-day curing period, the hydration products are unevenly distributed in the cement-treated soil, and the cement-treated soil particles do not fully interact with palm fiber. The evidence lies in the fact that when the damage occurs, a large number of palm fibers are directly pulled out in the cement-treated soil, so the contribution of palm fibers to the improvement of the compressive strength of cement-treated soil at this time is unstable.

Fig 8B shows the relationship between the unconfined compressive strength of the specimen and the palm fiber content at the curing age of 14 days. It can be seen in the figure that when 1%, 1.5% and 2% palm fiber are added to zeolite cement-treated soil, its strength is higher than 0% fiber. The strength of C15-Z0, C15-Z10 and C15-Z30 specimens increased with the increase of palm fiber content, and the peak compressive strength all appeared when the palm fiber content was 1.5%, and the compressive strength increased by 16%, 14% and 11% compared with the 0% sample, respectively. This time, there is a clear regularity between palm fiber content and strength. Compared with 7 days of curing age, palm fiber had a better effect on the strength increase of the specimen at 14 days. At this time, the hydration of cement is more sufficient, more hydrated calcium silicate gel is generated in cement-treated soil, the internal bonding of cement-treated soil is denser, and the combination of palm fiber, hydration products and soil particles is better, which can bear the load together. When damage occurs, the phenomenon of palm fibers being pulled out has been greatly reduced.

Fig 8C shows the relationship between the unconfined compressive strength of the specimen and the palm fiber content at the curing age of 28 days. It can be seen in the figure that the variation law of C15-Z0, C15-Z10, C15-Z30 specimens is similar to Fig 8B, all of which increase with the increase of palm fiber content, and the peak compressive strength also occurs when the palm fiber content is 1.5%, but its compressive strength does not increase much compared with 0% specimen. This may be related to the fact that the strength of the cement is fully utilized after 28 days of curing, thus exceeding the strength provided by the palm, and finally resulting in the fiber strength not being exerted. Comparing Fig 8A–8C, it can be seen that the strength varies greatly with the age of curing. From 7d to 28d, the C15-Z30 strength increased from 1.85 to 4.45MPa at a palm fiber content of 1.5, an increase of 141%. In this experiment, the optimal palm fiber incorporation was 1.5%, and the addition of too much palm fiber would cause the strength loss of cement-treated soil. From a microscopic point of view, too much palm fiber may cause direct contact between fiber and fiber in the mixture that almost don’t produce frictional force, rather than the contact between fiber and soil particles that can produce frictional force. This may be the main reason why the addition of too much palm fiber (2%) may decrease the strength.

### Deformation characteristics of the specimen

(1) Failure strain

Failure strain refers to the axial strain corresponding to palm fiber zeolite cement-treated soil reaching the peak of unconfined compressive strength, and its size is the key index to measure the brittleness and plasticity of cement-treated soil. In order to better measure the change in ductility of the specimen after incorporation of palm fiber, the concept of ductility ratio (DR) was introduced. The ductility ratio is defined as Eq (2).

$$DR=\frac{\Delta_{C-Z-F}}{\Delta_{C-Z}}$$ (2)

where:

Δ_C-Z-F_: Failure strain of zeolite cement-treated soil mixed with palm fiber;

Δ_C-Z_: Failure strain of zeolite cement-treated soil not mixed with palm fibers.

**Fig 9:**
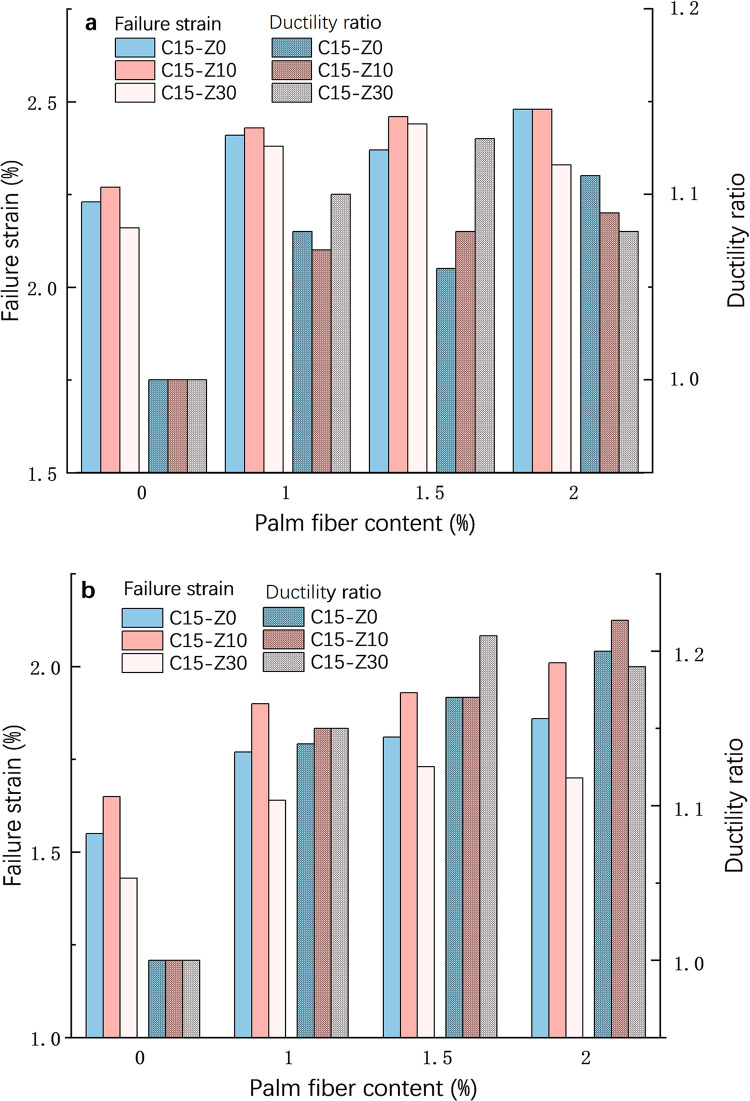
Relationship among failure strain, ductility ratio and palm fiber. a:7d, b:28d.

**Fig 9** shows the failure strain and ductility ratios of samples with different palm fiber content at 7 days (a) and 28 days (b). It can be seen from Fig 9A, 9B that with the increase of curing age, the failure strain of the specimen decreases to varying degrees, while the ductility ratio increases. Due to the insufficient interaction between cement, zeolite, palm fiber and soil particles during the 7d curing period, and its obvious irregularity, the test results at 28d were selected for analysis. As shown in Fig 9B, when the sample does not contain zeolite (C15-Z0), the failure strain increases monotonically with the palm fiber content, and it is at the 2% fiber content 19% higher than 0%, and the ductility ratio also gradually increases, and at the 2% fiber content the ductility ratio is 1.2 (1 at 0% fiber content). When the zeolite content is 10% (C15-Z10), the failure strain increases monotonically with the palm fiber content, and at the 2% fiber content, it is 21% higher than 0%, and the ductility ratio is also increased, and at the 2% fiber content the ductility ratio is 1.23 (1 at 0% fiber content). When the zeolite content is 30% (C15-Z30), the situation is different. When the palm fiber content is less than or equal to 1.5%, the change law is the same as before. However, when the palm fiber is 2%, both the failure strain and the ductility ratio decrease (compared to that at 1.5% palm fiber), which may be related to the excessive zeolite content that complicates the action between them.

(2) Elastic modulus

For elastic materials, the modulus of elasticity is defined as the stress in the unidirectional stress state of the material divided by the strain in that direction, and its magnitude is the stress value required for the elastic deformation of the material per unit. It is an indicator of the difficulty of elastic deformation of materials, the greater the modulus of elasticity, the greater the stiffness of the material, the less likely it is to deform. Cement-treated soil is a non-elastic material, and the elastic modulus is generally defined as the secant modulus of cement-treated soil at 50% peak strength, termed as E_50_, and then E_50_ is used to characterize the elastic modulus of palm fiber zeolite cement-treated soil. The definition of E_50_ is shown in Eq (3).

$$E_{50}=\sigma /2\varepsilon_{0.5}$$ (3)

where:

E_50_: the elastic modulus of palm fiber zeolite cement-treated soil;

σ: Peak stress of palm fiber zeolite cement-treated soil;

ε_0.5_: The strain corresponding to 50% of the peak stress.

**Fig 10:**
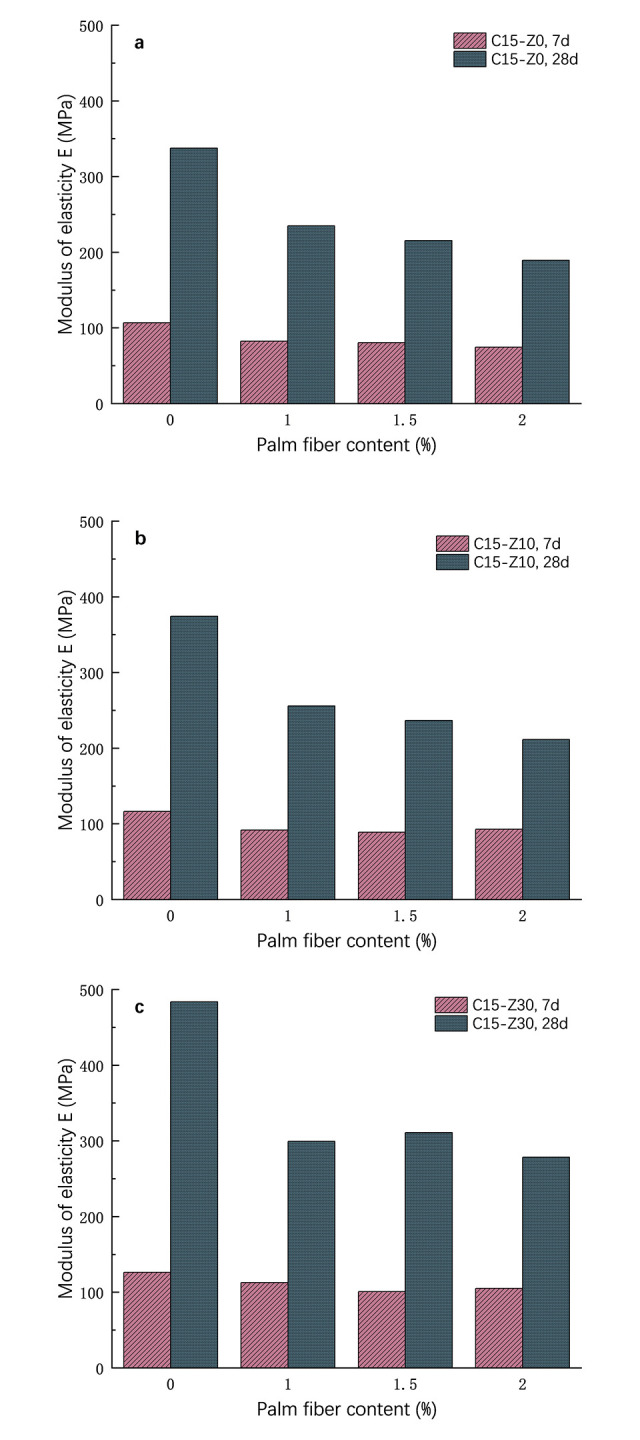
Elastic modulus of the specimen. Zeolite substitution rate. a: 0%, b: 10%, c: 30%.

Fig 10A–10C were elastic modulus at 15% cement content, with a replacement rate of 0%, 10% and 30%, for curing periods of 7 d and 28 d, respectively. On the whole, the 7d elastic modulus of zeolite cement-treated soil with the same palm fiber content is less than that of 28 d, and the elastic modulus increases with the increase of curing age. Fig 10A shows the elastic modulus of zeolite content at 0% and different palm fiber content. Under the 7-day curing period, the elastic modulus of cement-treated soil at 0%, 1%, 1.5% and 2% palm fiber content was 106.8MPa, 82.6MPa, 80.6MPa and 74.6MPa, respectively. At the 28-day curing period, they were 337.6 MPa, 234.9 MPa, 215.4 MPa and 189.5 MPa, respectively. The elastic modulus of plain cement-treated soil was the largest, and it was 3.18 times that of the 7-day curing period. For the 2% palm content, its modulus of elasticity is 2.52 times that of the 7-day modulus. Fig 10B shows the elastic modulus of zeolite content of 10%, with different palm fiber content. Under the 7-day curing period, the elastic modulus of cement-treated soil at 0%, 1%, 1.5% and 2% palm fiber content was 116.6MPa, 92.0MPa, 89.1MPa and 93MPa, respectively. At the 28-day curing period, they were 374.5 MPa, 255.8 MPa, 236.7 MPa and 211.4 MPa, respectively. Compared with the case of 0% zeolite, the modulus of elasticity increases to a certain extent. Fig 10C shows the modulus of elasticity at 30% zeolite content with different palm fiber content. Under the 7-day curing period, the elastic modulus of cement-treated soil at 0%, 1%, 1.5% and 2% palm fiber content was 126.4MPa, 112.7MPa, 101.0MPa and 105.2MPa, respectively. At the 28-day curing period, they were 483.9MPa, 299.4MPa, 311.1MPa and 278.6MPa, respectively. Compared with the case of 0% zeolite, the modulus of elasticity has a large increase. However, they all gradually decrease with the increase of palm fiber.

In summary, after adding palm fiber in zeolite cement-treated soil, the failure strain of the sample becomes large, and the failure strain decreases with the increase of curing age. And when other conditions are certain, the addition of palm fiber can reduce the elastic modulus E_50_ of cement-treated soil and reduce the stiffness. The modulus of elasticity for specimens with 30% zeolite replacement rate is greater than 10% zeolite replacement rate at the same palm fiber content. The addition of fibers increases the ductility of the specimen and reduces the brittleness, which is of great significance for engineering seismic resistance.

### Multiple regression analysis

Regression analysis is a statistical analysis method that determines the quantitative relationship between two or more variables that depend on each other. It mainly analyzes the statistical relationship between things, finds the change law between independent variables and response variables, and describes and reflects this relationship in the form of regression equations, helping to accurately grasp the degree of influence of each independent variable on the corresponding variable, and providing a reliable reference for scientific prediction. This section selects cement-treated soil with 15% cement content and conducts multiple regression analysis on its strength under different curing ages, zeolite replacement rate and palm fiber content, and obtains multiple regression equations for data prediction and practical application of engineering.

After observing the experimental data, the nonlinear surface fitting was selected to reflect the relationship between the strength of palm fiber zeolite cement-treated soil and the respective variable factors, and the fitting function is shown in Eq (4).

$$z=z_{0}+ax+by+cx^{2}+dy^{2}+fxy$$ (4)

where:

z: Unconfined compressive strength (MPa) of palm fiber zeolite cement-treated soil

x: Zeolite replacement rate%;

y: Palm fiber content%;

z0、a、b、c、d、f: Relevant parameters.

In the multiple regression fitting analysis, it was discussed according to curing age.

#### Seven days curing period

**Fig 11:**
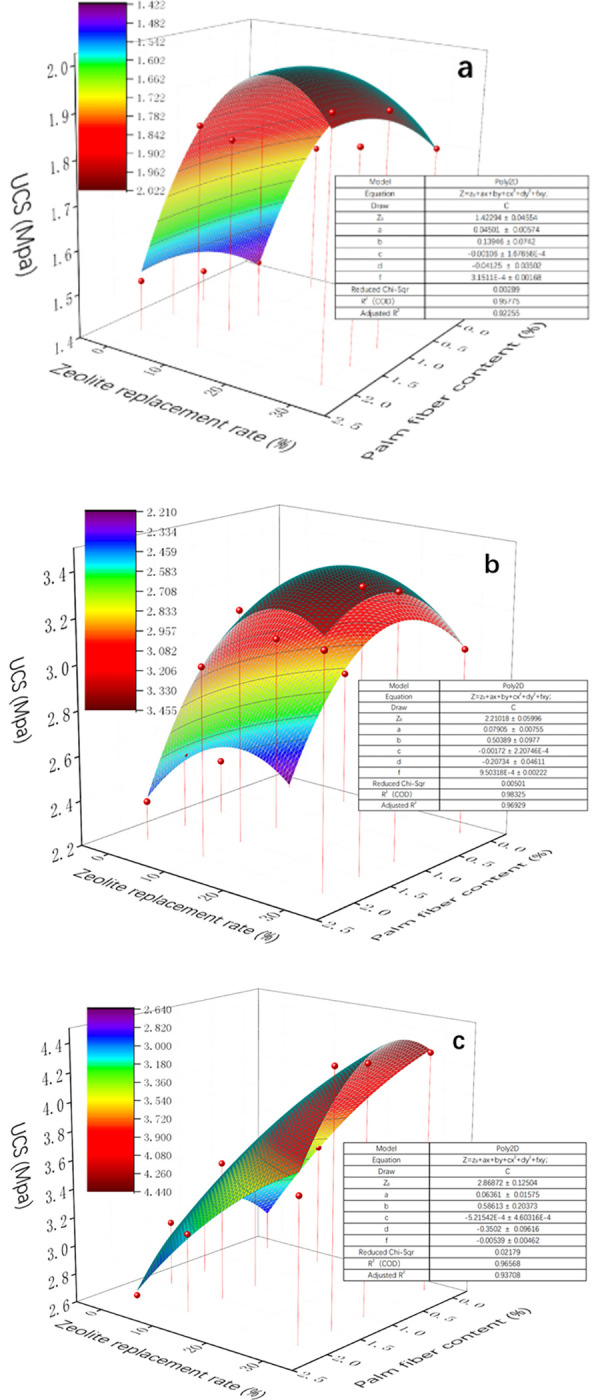
Fitted surface diagram of UCS of specimen. a:7d, b:14d, c:28d.

Fig 11 A shows the specimen fitting surface of the compressive strength at a curing age of 7 days. It can be seen from the figure that the whole surface increases with the increase of zeolite substitution rate, and the surface between the various palm fiber content is relatively smooth. The coefficient of determination of the surface R^2^ is 0.958 and the adjusted R^2^ is 0.923, which indicates that 92.3% of the dependent variable can be explained by the fitted surface, which has good fitting results. The parameter values of the regression model are given in Fig 11A and Table 7, and the regression equation for the 7-day compressive strength of palm fiber zeolite cement-treated soil can be obtained by substituting the parameter values into the fitted surface function.

**Table 7:** Quadratic regression model parameter values.

parameter	value	std. error	t value	Prob.>|t|	correlation
z0	1.42294	0.04554	31.2448	7.13923E-8	0.88384
a	0.04501	0.00574	7.84805	2.26274E-4	0.97803
b	0.13946	0.0742	1.8795	0.10923	0.97586
c	-0.00108	1.67656E-4	-6.44957	6.58032E-4	0.96864
d	-0.04125	0.03502	-1.17783	0.28346	0.96439
f	3.1511E-4	0.00168	0.18724	0.85764	0.8592

#### Fourteen days curing period

Fig 11B shows the compressive strength fitting surface of palm fiber zeolite cement-treated soil at a curing age of 14 days. As can be seen from the figure, the entire surface rises with the increase of zeolite replacement rate, and the curved surface appears rounded from the perspective of palm fiber content. The coefficient of determination of the surface R2 is 0.983 and the adjusted R2 is 0.969, which indicates that 96.9% of the dependent variable can be explained by the fitted surface, which has a good fit result. The parameter values of the regression model are given in Fig 11B and Table 8, and the regression equation for the 14-day compressive strength of palm fiber zeolite cement-treated soil can be obtained by substituting the parameter values into the fitted surface function.

**Table 8:** Quadratic regression model parameter values.

parameter	value	std. error	t value	Prob.>|t|	correlation
z0	2.21018	0.05996	36.85902	2.66083E-8	0.88384
a	0.07905	0.00755	10.46742	4.46115E-5	0.97803
b	0.50389	0.0977	5.1575	0.0021	0.97586
c	-0.00172	2.20746E-4	-7.78943	2.35852E-4	0.96864
d	-0.20734	0.04611	-4.49638	0.00412	0.96439
f	9.50318E-4	0.00222	0.42888	0.68298	0.8592

#### Twenty-eight days curing age

Fig 11C shows the fitting surface of the compressive strength of palm fiber zeolite cement-treated soil at a curing period of 28 days. It can be seen from the figure that the whole surface increases with the increase of zeolite substitution rate, and the phenomenon of first increasing and then decreasing with the increase of palm fiber content. The coefficient of determination of the surface R2 is 0.966 and the adjusted R2 is 0.937, which indicates that 93.7% of the dependent variable can be explained by the fitted surface, which has good fitting results. The parameter values of the regression model are given in Fig 11C and Table 9, and the regression equation for the 28-day compressive strength of palm fiber zeolite cement-treated soil can be obtained by substituting the parameter values into the fitted surface function.

**Table 9:** Quadratic regression model parameter values.

parameter	value	std. error	t value	prob. >|t|	correlation
z0	2.86872	0.12504	22.94251	4.49298E-7	0.88384
a	0.06361	0.01575	4.03945	0.00681	0.97803
b	0.58613	0.20373	2.87699	0.02817	0.97586
c	-5.21542E-4	4.60316E-4	-1.13301	0.30045	0.96864
d	-0.3502	0.09616	-3.642	0.01081	0.96439
f	-0.00539	0.00462	-1.16569	0.28798	0.8592

Cement, as the preferred material for soil solidification, has been widely used, yet its production contributes significantly to greenhouse gas emissions due to being one of the major sources of carbon emissions. Zeolite, a natural material with pozzolanic activity, can react with hydrated lime and water to produce substances with excellent gelling properties when ground into powder. Therefore, a strategy of partially replacing cement with zeolite powder is proposed, aiming to reduce cement consumption and consequently decrease carbon dioxide emissions. This approach is not only environmentally friendly but also practical. More importantly, this substitution method can effectively enhance the strength of solidified materials, which has profound implications for advancing the ambitious goals of "carbon peak" and "carbon neutrality."

Furthermore, palm fiber, as a low-cost and easily accessible plant fiber material, possesses green and environmentally friendly characteristics, making it promising for widespread application in the field of civil engineering. Incorporating palm fibers as reinforcement into the soil not only significantly enhances the ductility of the soil but also effectively reduces the risk of brittle failure by increasing the frictional force between soil particle and fiber. This innovative application holds considerable practical value for improving the disaster prevention and mitigation capabilities of civil engineering projects.

## Conclusions

Our research results showed that both cement and zeolite exhibit significant effects in soil improvement, specifically in significantly enhancing the unconfined compressive strength of soil. More importantly, after adding palm fibers, it not only increases the soil’s strength but also exhibits a unique toughening effect during the soil failure stage, significantly improving the soil’s strain capacity. This point undoubtedly deserves further research and attention. The dominant conclusions of this paper are as follows:

1. The higher the cement content in cement-treated soil and the longer the curing period, the greater the strength. At 30% zeolite content, the specimen has the maximum unconfined compressive strength.
2. The replacement rate of equal strength zeolite is about 40%. Below this value, the strength change rate is positive, otherwise negative.
3. In the experiment of zeolite replacing cement, regardless of the cement and zeolite content, the greater the peak strength of the specimen, the smaller the failure strain.
4. The optimal palm fiber content is about 1.5%, at this time the unconfined compressive strength is maximum. In addition, the greater the content of palm fiber, the higher the failure strain and ductility ratio. Conversely, its elastic modulus tends to decrease with the content of palm fiber.
5. The multiple regression equation can be used to predict the effect of zeolite and palm fiber on the unconfined compressive strength of cement-treated soil, and 90% of strength change can be interpreted.
